# Perioperative impact of ultrasound-guided ilioinguinal and iliohypogastric nerve blocks in patients undergoing pelvic fracture surgery

**DOI:** 10.1097/MD.0000000000038634

**Published:** 2024-06-28

**Authors:** Jie Shen, Hui Ma, Xiaohui Yang, Mingcan Hu, Jieyin Tian

**Affiliations:** a Department of Anesthesiology, Handan First Hospital, Handan, Hebei Province, China.

**Keywords:** ilioinguinal nerve block, inguinal hernia nerve block, internal fixation surgery, pelvic fractures, ultrasound-guided anesthesia

## Abstract

Pelvic fractures present a severe and complex clinical challenge. This study aimed to compare ultrasound-guided ilioinguinal (IIN) and iliohypogastric nerve (IHN) blocks with conventional general anesthesia (GA) in patients undergoing internal fixation surgery for pelvic fractures. A retrospective analysis was conducted on 100 patients equally divided into ultrasound-guided and control groups. The study monitored hemodynamics, intraoperative anesthesia drug usage, postoperative pain levels, and the incidence of adverse reactions between the 2 groups. The ultrasound-guided group underwent ultrasound-guided IHN and IIN blocks combined with GA. The ultrasound-guided group exhibited significant advantages for hemodynamic measurements at specific time points, lower consumption of Propofol and Remifentanil, and reduced pain intensity across all evaluated time intervals (*P* < .05). The incidence rate of adverse reactions was significantly lower in the ultrasound group (*P* = .016). Ultrasound-guided anesthesia is a superior alternative to conventional GA for managing pelvic fractures through internal fixation surgery. It offers advantages in terms of hemodynamic stability, drug consumption, postoperative pain management, and adverse reaction reduction.

## 1. Introduction

Pelvic fractures, commonly caused by severe trauma, are widespread and can have severe consequences, including a significant risk of illness and death.^[1]^ The complex and fragile structure of the pelvis, which is closely linked to various organ systems including the gastrointestinal, urinary, and arterial systems, greatly complicates the therapeutic treatment of severe fractures. The simultaneous presence of pelvic fractures alongside other physical injuries, such as visceral damage and multiple fractures in the extremities or other skeletal areas, contributes to the intricacy of therapy. In the initial phases of pelvic fracture, there is a high likelihood of significant bleeding which can result in life-threatening consequences such as shock, metabolic acidosis, coagulopathy, and in severe instances, death.^[2,3]^ The considerable blood loss that is linked with this condition is well-known for causing major changes in the circulatory system, which require careful consideration while choosing an anesthetic. The mentioned consideration is not only related to procedures, but it also has a significant and deep connection with both immediate surgical results and long-term prognosis. This highlights the importance of an anesthetic approach that is specifically designed for the complex difficulties of pelvic fractures and is in line with the most advanced medical standards.^[4]^

Pelvic fractures are frequently treated with internal fixation surgery. However, the conventional approach of using general anesthesia (GA) with tracheal intubation has presented several difficulties.^[5]^ These factors encompass extended periods of anesthesia, unpredictable changes in blood flow, and potentially less-than-ideal effects of anesthesia. The introduction of ultrasound-guided ilioinguinal (IIN) and iliohypogastric nerve (IHN) blocks has brought about a significant change in the field. These blocks provide the ability to visualize peripheral nerves in real-time, enhance the accuracy of anesthetic administration, minimize disruptions to the body systems, and allow for precise targeting of specific tissues.^[6]^ These benefits not only improve patient stability, but also lead to a more regulated and effective surgical experience. Ultrasound-guided nerve blocks represent significant progress in surgical anesthetic, placing themselves as a promising option with the potential to enhance surgical outcomes, patient comfort, safety, and overall healthcare efficiency.^[7,8]^ Nevertheless, the whole ramifications of the approach still necessitate additional investigation and verification through rigorous clinical research and trials.

The findings of this study have the capacity to fundamentally transform the discipline of anesthesia in orthopedic procedures. This research has the potential to establish a new standard of patient treatment by showcasing the effectiveness, safety, and advantages of ultrasound-guided nerve blocks. This could provide additional guidance to doctors in making well-informed decisions, enabling them to implement tailored treatment options that are supported by evidence. Moreover, the results of this study have the potential to stimulate other investigations in several domains of anesthetic care and pain management. This underscores the significance of ongoing innovation and research in order to better surgical results, diminish complications, and eventually enhance the quality of life for patients with serious injuries.

## 2. Materials and methods

### 2.1. Study design

**Figure 1.**
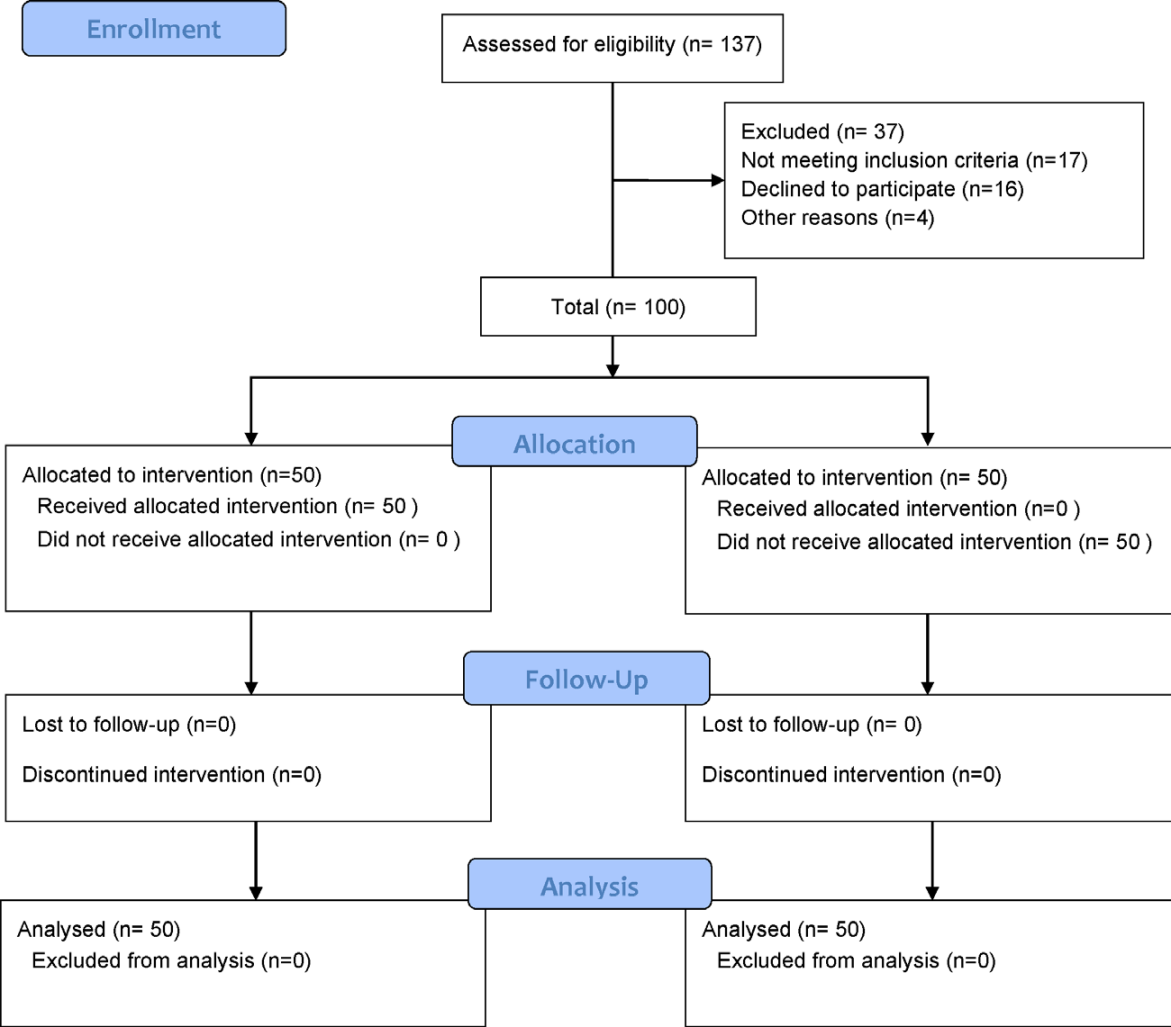
Flow diagram of the study design and population.

A retrospective analysis was conducted on 100 patients who underwent internal fixation surgery for pelvic fractures at Handan First Hospital from January 2019 to December 2022. The patients were equally divided into 2 groups: an ultrasound-guided group (n = 50) and control group (n = 50) (Fig. 1). All participants provided informed consent and the study protocol was approved by the Ethics Committee of Handan First Hospital.

### 2.2. Inclusion and exclusion criteria

Inclusion criteria consisted of a confirmed diagnosis of a pelvic fracture through CT scans or other medical examinations, an anesthesia classification marked by the American Society of Anesthesiologists as Grade II-III, a signed informed consent form, and willingness to accept internal fixation surgical treatment. Conversely, the exclusion criteria were allergy to the research drugs involved in the study, significant organ dysfunction, incomplete clinical data, existing mental illness, inability to tolerate surgical treatment, and the presence of contraindications related to anesthesia. These criteria serve as essential guidelines to ensure the selection of appropriate subjects for research, eliminating potential biases and confounding factors, thus enhancing the validity and reliability of the study.

### 2.3. Anesthesia procedure

Both groups were instructed to fast from water for 8 hours and from food for 12 hours, preoperatively. Thirty minutes prior to surgery, an intramuscular injection of atropine (0.5 mg) and 0.1 g of phenobarbital sodium were administered. Open venous access was established, with routine monitoring of electrocardiogram, blood pressure, and blood oxygen saturation.

In the ultrasound group, the patients underwent ultrasound-guided IHN and IIN block combined with GA. A Venue 50 high-frequency linear ultrasound probe (General Electric Company) was positioned vertically on the affected side anterior superior iliac spine and abdominal wall, parallel to the plane connecting the umbilicus and anterior superior iliac spine. The external oblique, transversus abdominis, and internal oblique muscles were identified by recognizing the echo shadow between the transversus abdominis and internal oblique muscles, thereby locating the target nerves. Three distances were measured: skin to the IHN and IIN, distance between the IIN and IHN, and distance from the anterior superior iliac spine to the IIN. After disinfection of the puncture point, a needle was inserted from outside to inside in the plane, withdrawing upon approach to the echo shadow to confirm no blood, followed by injection of 15 mL of 0.5% ropivacaine (Shijiazhuang No.4 Pharmaceutical, batch number:201708), observing the diffusion after the injection. GA was induced by intravenous injection of propofol 1 to 1.5 mg/kg, sufentanil 0.3 µg/kg, and rocuronium 0.6 mg/kg. After tracheal intubation, the patient was connected to an anesthesia machine for mechanical ventilation. Anesthesia maintenance included inhalation of sevoflurane (1%–2%), continuous intravenous infusion of remifentanil 0.1 to 0.2 µg/(kg·min)), dexmedetomidine 0.4 µg/(kg·h)), and administration of 2 mg rocuronium every 45 minutes until the conclusion of the surgery.

The control group underwent conventional GA with induction and maintenance drugs, administration method, and dosage consistent with those of the ultrasound group.

### 2.4. Observation indicators and evaluation criteria

The 2 groups of patients were compared at different time points in terms of hemodynamics, intraoperative anesthesia drug usage, postoperative pain levels in the surgical area, and incidence of adverse reactions. Hemodynamics: The hemodynamics of both groups were compared at various time intervals, including 5 minutes before surgery commencement (T0), at surgery initiation (T1), 30 minutes into surgery (T2), 60 minutes into surgery (T3), end of surgery (T4), and 15 minutes after extubation following general anesthesia (T5). The specific hemodynamic parameters assessed were mean arterial pressure (MAP) and heart rate (HR). Intraoperative Anesthesia Drug Usage: The consumption levels of specific anesthesia medications, including remifentanil, propofol, sufentanil, and rocuronium, were recorded and compared between the 2 groups. Postoperative Pain Levels in the Surgical Area: The intensity of postoperative pain in the surgical area was compared between the 2 groups at 2, 12, 24, and 48-hours post-surgery. Pain levels were evaluated using the visual analog scale (VAS), with scores ranging from 0 to 10. A higher score indicated a more severe pain level. Adverse Reactions: The incidence of adverse reactions, such as postoperative restlessness, skin itchiness, nausea, and vomiting, was documented and compared between the 2 groups.

### 2.5. Statistical analysis

In the analysis of the study data, SPSS 21.0 statistical software was employed. For continuous data, the mean ± standard deviation was used as a measure of central tendency and dispersion, respectively, and comparisons between groups were conducted using Student *t* test. This allowed for the assessment of differences in means between the 2 independent groups. For categorical data, proportions were used for representation, and the chi-square (χ²) test was used to compare the groups and identify relationships or associations between categorical variables. A *P* value <.05 was considered to indicate a statistically significant difference, providing a standard threshold for determining the probability that the observed difference occurred by random chance.

## 3. Results

### 3.1. Participant analysis

We retrospectively analyzed 100 patients who underwent internal fixation surgery for pelvic fractures. The ultrasound-guided Group: Included 23 females and 27 males, aged between 41 and 75 years (average 58.32 ± 2.14). Injury causes were classified as falls from a height in 15 cases, traffic accidents in 22 cases, and crushing injuries in 13 cases. Fracture types included acetabular anterior column fractures (15) and pubic branch fractures (35). Time from injury to surgery ranged from 1 to 12 days, averaging 3.12 ± 0.21 days, with BMI values ranging from 19 to 26 kg/m^2^ (average 23.01 ± 1.22 kg/m^2^). The control group consisted of 22 females and 28 males, aged between 42 and 78 years (average 58.51 ± 2.04). Injury etiologies included falls from height in 17 cases, traffic accidents in 24 cases, and crushing injuries in 9 cases. Fracture types were similar to those in the ultrasound group, with no statistically significant difference between the groups (*P* > .05), making them comparable.

### 3.2. Comparative hemodynamic analysis

At the designated time points (T0, T1, T2, T3, T4, T5), hemodynamic measurements revealed a significant decrease in MAP and HR at T1 and T2 in the ultrasound-guided group compared to the control group (*P* < .05). The control and ultrasound groups exhibited distinct hemodynamic responses, possibly reflecting the physiological effects of the ultrasound-assisted procedure. In contrast, the remaining time intervals (T0, T3, T4, and T5) showed no statistically significant variations, highlighting the consistency and homogeneity of the measurements beyond the early stages of the procedure (Table 1).

**Table 1:** Comparative analysis of mean arterial pressure at various surgical time points between conventional anesthesia and ultrasound-guided groups.

MAP (mm Hg)	Conventional anesthesia group (n = 50)	Ultrasound-guided anesthesia group (n = 50)	t-value	*P* value
T0 (5 min before surgery)	84.22 ± 9.03	82.98 ± 9.08	0.029	.978
T1 (surgery initiation)	69.12 ± 11.12	81.91 ± 9.02	6.400	.000
T2 (30 min into surgery)	71.33 ± 7.97*	83.72 ± 8.51	6.489	.000
T3 (60 min into surgery)	80.18 ± 8.98	82.56 ± 8.99	1.192	.235
T4 (end of surgery)	81.05 ± 7.75	82.91 ± 8.77	0.810	.425
T5 (15 min post-extubation)	82.05 ± 9.01	84.12 ± 8.69	0.260	.805

MAP = mean arterial pressure, measured in millimeters of mercury (mm Hg). Time points from T0 to T5 are defined to assess hemodynamic stability throughout the surgical procedure. A *P* value < .05 indicates a statistically significant difference between groups.

### 3.3. Analysis of HR between control and ultrasound groups

Table 2 presents the HR measurements at specified surgical time points (T0, T1, T2, T3, T4, and T5) for both the control group and the group that underwent ultrasound-guided IHN and IIN blocks in combination with GA. At T1 and T2, there was a statistically significant decrease in HR in the ultrasound-guided group compared to the control group (*P* < .05), demonstrating the efficacy of the ultrasound-guided nerve blocks in stabilizing HR during these critical early stages of surgery. At other measured time points (T0, T3, T4, T5), no significant differences were observed in HR between the 2 groups, indicating a consistent hemodynamic response across both groups for the remainder of the procedure.

**Table 2:** Comparison of heart rate at various time points between conventional anesthesia and ultrasound-guided anesthesia groups.

Time Point	Conventional anesthesia group (n = 50)	Ultrasound-guided anesthesia group (n = 50)	t-value	*P* value
T0 (5 min before surgery)	85.88 ± 7.23	85.60 ± 7.54	0.006	.993
T1 (surgery initiation)	78.11 ± 6.62	82.89 ± 7.59	3.056	.003
T2 (30 min into surgery)	79.66 ± 6.55	83.14 ± 7.65	2.423	.019
T3 (60 min into surgery)	82.53 ± 7.49	84.77 ± 7.89	1.008	.320
T4 (end of surgery)	83.62 ± 7.42	84.88 ± 7.59	0.912	.346
T5 (15 min post-extubation)	86.05 ± 6.71	86.21 ± 8.16	0.110	.910

HR is presented as heart rate, measured in beats per minute (beat/min). Time points from T0 to T5 are defined to assess the heart rate response at critical phases of the surgical procedure. A *P* value < .05 indicates a statistically significant difference between groups.

### 3.4. Analysis of anesthetic drug usage between control and ultrasound groups

The analysis in Table 3 details the usage of anesthetic drugs such as propofol, Remifentanil, Sufentanil, and Rocuronium Bromide among the groups. This assessment showed a statistically significant decrease in the usage of Propofol and Remifentanil in the ultrasound-guided group compared to the control group (*P* < .05), reflecting the differential anesthesia protocols and effectiveness in reducing drug consumption. In contrast, the usage of Sufentanil and Rocuronium Bromide showed no significant changes between the groups (*P* > .05), indicating uniform administration of these drugs across both groups.

**Table 3:** Comparative analysis of anesthetic drug usage between conventional anesthesia and ultrasound-guided anesthesia groups.

Group	Propofol (mg)	Remifentanil (µg)	Rocuronium bromide (mg)	Sufentanil (µg)
Conventional anesthesia group (n = 50)	570.10 ± 63.77	1.07 ± 0.31	9.92 ± 0.86	37.49 ± 5.88
Ultrasound-guided anesthesia group (n = 50)	443.01 ± 47.12	0.76 ± 0.22	10.09 ± 0.86	34.49 ± 5.88
t-value	11.83	6.106	0.356	0.658
*P* value	.00	.00	.80	.68

### 3.5. Evaluation of postoperative pain reduction between control and ultrasound groups

The statistical analysis indicated a significant reduction in postoperative pain levels at all evaluated time points (*P* < .05) in the ultrasound group compared to the control group. This reduction across the time intervals highlights the effectiveness of the ultrasound-guided interventions in managing postoperative pain. The statistically significant t-values confirm the therapeutic benefits of ultrasound techniques in enhancing postoperative comfort (Table 4).

**Table 4:** Comparison of postoperative pain levels at different time intervals between conventional anesthesia and ultrasound-guided anesthesia groups.

Group	Postoperative 2h	Postoperative 48h	Postoperative 12h	Postoperative 24h
Conventional anesthesia group (n = 50)	7.58 ± 1.33	4.46 ± 1.16	2.66 ± 0.35	2.21 ± 0.53
Ultrasound-guided anesthesia group (n = 50)	6.81 ± 1.30	3.16 ± 0.82	2.29 ± 0.27	1.86 ± 0.28
t-value	6.94	4.36	6.78	6.27
*P* value	.00	.00	.00	.00

### 3.6. Analysis of adverse reactions between control and ultrasound groups

A salient observation was the considerable reduction in the overall incidence rate of adverse reactions in the ultrasound group, with an aggregate incidence of 4 (8.00%) compared to 14 (28.00%) in the control group. This underscores the benefits of ultrasound-guided protocols, with itching reported to be less frequent in the ultrasound group and lower or equivalent rates of both nausea and vomiting. Notably, agitation was completely absent in the ultrasound group, reflecting substantial improvement in patient comfort. These findings were further strengthened by a χ²-value of 5.488 and a *P* value of .0191, confirming the significance of the differences observed. By significantly mitigating the adverse reactions commonly associated with postoperative care, ultrasound guidance appears to offer a more patient-centered approach (Table 5).

**Table 5:** Comparative analysis of adverse reaction incidence rates between conventional anesthesia and ultrasound-guided anesthesia groups.

Group	Skin itching (%)	Nausea (%)	Vomiting (%)	Agitation (%)	Total incidence (%)
Conventional anesthesia group (n = 50)	3 (6.00)	2 (4.00)	4 (8.00)	5 (10.00)	14 (28.00)
Ultrasound-guided anesthesia group (n = 50)	1 (2.00)	2 (4.00)	1 (2.00)	0 (0.00)	4 (8.00)
χ²-value					5.488
*P* value					.0191

## 4. Discussion

Pelvic fractures, comprising a mere 2% of clinical fractures, are urgent medical crises due to the intricate structure of the pelvis, essential arterial networks, neurological processes, and organs. The fractures are significant due to their potential to cause immediate severe consequences, resulting in disability rates ranging from 50% to 60% and fatality rates as high as 10%.^[3,9]^ The management of pelvic fractures is comprehensive, involving prompt emergency care, possible surgical intervention, and extensive rehabilitation. The intricate nature of the injuries, possibility of accompanying trauma, and urgency for prompt and thorough treatment pose considerable obstacles in contemporary trauma care.^[10,11]^

Administering anesthesia during pelvic fracture surgery is a distinct and complex difficulty. Traditional GA is frequently employed, providing transient suppression of the central nervous system and alleviation of pain.^[12]^ Nevertheless, the partial effectiveness of GA in preventing intraoperative stimulation has several consequences. While it efficiently reduces surgical pain, it does not fundamentally suppress stress responses, resulting in different levels of fluctuation in blood flow. This can have a negative impact on the patient recuperation after surgery, putting stress on the cardiovascular system. Furthermore, GA might lead to adverse side effects such as aspiration, intraoperative agitation, and postoperative nausea.^[13]^ The problems associated with pelvic fracture surgery can vary in severity and scope, making it difficult to treat. This highlights the importance of developing more accurate and targeted anesthetic procedures that are specifically designed to meet the special demands of this type of surgery.^[14,15]^ Continuous research and developments in diagnostic procedures, surgical methods, and holistic care are crucial for improving outcomes. One unique example is the use of ultrasound-guided IIN and IHN blocks in pelvic fracture surgeries.

Our study revealed that patients in the ultrasound group maintained stable MAP and HR at different time intervals, unlike those in the control group (*P* < .05). This stability can be attributed to the precision of the ultrasound-guided approach in nerve targeting, minimizing sympathetic nervous system involvement, and ensuring circulatory steadiness. Significantly lower consumption of Propofol and Remifentanil in the ultrasound group indicated efficient anesthesia with reduced drug requirements (*P* < .05). This implies cost-effectiveness and potential reduction in anesthetic-related complications. The ultrasound group also exhibited lower postoperative pain scores and fewer adverse reactions (*P* < .05). This emphasizes the superiority of the ultrasound-guided approach for pain management, thus facilitating better patient recovery and satisfaction.

These research findings accentuate the benefits of ultrasound-guided IIN and IHN blocks, shedding light on the underlying mechanisms that make them effective for pelvic fracture surgery. By employing ultrasound guidance, clinicians can gain clear visualization of the target nerves, including their surrounding anatomical structures, leading to precise localization and direct injection into the nerve sheath.^[16]^ This accuracy not only shortens the onset time of anesthesia but also reduces the quantity required, thereby limiting the impact on sympathetic nerves. Consequently, this promotes circulatory stability and avoids vasodilation.^[17,18]^ Furthermore, ultrasound-guided blocks enable comprehensive real-time observation of the injection and diffusion processes of anesthetic drugs, thereby negating the potential risks associated with unpredictable anatomical variations. This precise control ensures adequate diffusion around the target nerves, enhancing the overall quality of anesthesia and contributing to a safer and more effective surgical experience.^[7]^

Our study revealed significant differences in hemodynamic responses and pain management between the ultrasound-guided and control groups during pelvic fracture surgeries. Specifically, we observed a significant decrease in MAP at critical surgical stages (T1 and T2) in the ultrasound group (*P* < .05), indicating enhanced hemodynamic stability, likely attributed to the effective pain control achieved through ultrasound-guided nerve blocks. Conversely, the ultrasound group exhibited an increase in HR during these phases (*P* < .05), suggesting a distinct physiological response to the anesthetic technique. Importantly, the ultrasound-guided approach resulted in significantly lower postoperative pain scores across all time intervals (*P* < .05), demonstrating its efficacy in pain management and reducing the reliance on systemic analgesics such as Propofol and Remifentanil. Additionally, a significant reduction in post-surgical adverse reactions was noted in the ultrasound group (*P* = .016), including decreased incidences of skin itching, nausea, and vomiting, thereby enhancing patient comfort and satisfaction. These findings emphasize the clinical benefits of ultrasound-guided blocks in pelvic fracture surgeries, providing improved hemodynamic stability and pain control, while minimizing adverse reactions.

Previous research has laid the groundwork for our study, offering insights and perspectives that have enriched our understanding. Lao et al^[19]^ focused on post-surgical complications in the ilioinguinal approach, particularly regarding muscle morphology. Our study builds on this by highlighting the benefits of ultrasound-guided nerve blocks in pelvic fractures, including reduced pain and adverse reactions. Yang et al^[20]^ comparison of surgical approaches for pelvic fractures parallels our findings on the advantages of ultrasound-guided anesthesia in reducing operative time, blood loss, and enhancing postoperative recovery. Huh et al^[21]^ work on ultrasound-guided nerve blocks in hip hemiarthroplasty corroborates our findings on the effectiveness of these blocks in pain management. Our research extends these concepts, demonstrating the broad applicability and benefits of ultrasound-guided techniques in various stages of pelvic fracture surgery, underscoring their importance in modern orthopedic procedures.

This study, while offering valuable insights, has several limitations. Firstly, our retrospective design limited the standardization of pain assessment and anesthesia depth. Baseline VAS scores were retrospectively assessed from medical records, potentially overlooking variability in trauma mechanisms and injury severities. Additionally, the lack of systematic evaluation of patients’ recovery status may affect the reliability of postoperative pain assessments. Our focus was primarily on the intraoperative and immediate postoperative periods, limiting long-term pain management insights. Important factors like patients’ opioid history, additional injuries, and the absence of sensory level assessments pre-anesthesia were not comprehensively evaluated. The single-institution setting may limit generalizability, and potential biases inherent in such a study could affect broader applicability.

Future research should incorporate standardized methods for assessing anesthesia depth, baseline VAS scores, and recovery status to provide a more accurate and complete picture of anesthetic management in pelvic fracture surgeries. Prospective studies are needed to address these limitations and enhance our understanding of the effectiveness of ultrasound-guided anesthesia.

## 5. Conclusions

In conclusion, the utilization of ultrasound-guided IHN and IIN blocks alongside GA during pelvic fracture surgery seems to improve surgical safety, decrease the need for anesthetic medications, sustain stable blood flow, and lessen postoperative discomfort in patients. Nevertheless, comprehensive research is required to validate these findings.

## Acknowledgments

We appreciate the cooperation and informed consent provided by the patients in this study.

## Author contributions

**Data curation:** Xiaohui Yang, Jieyin Tian.

**Formal analysis:** Hui Ma.

**Methodology:** Jie Shen.

**Resources:** Mingcan Hu.

**Software:** Hui Ma.

**Validation:** Jieyin Tian.

**Writing – original draft:** Jie Shen.

**Writing – review & editing:** Jie Shen.
